# Molecular Epidemiology of Oropouche Virus, Brazil

**DOI:** 10.3201/eid1705.101333

**Published:** 2011-05

**Authors:** Helena Baldez Vasconcelos, Márcio R.T. Nunes, Lívia M.N. Casseb, Valéria L. Carvalho, Eliana V. Pinto da Silva, Mayra Silva, Samir M.M. Casseb, Pedro F.C. Vasconcelos

**Affiliations:** Author affiliations: Instituto Evandro Chagas, Ananindeua, Brazil (H. Baldez Vasconcelos, M.R.T. Nunes, L.M.N. Casseb, V.L. Carvalho, E.V. Pinto da Silva, M. Silva, S.M.M. Casseb, P.F.C. Vasconcelos);; Universidade do Estado do Pará, Belém, Brazil (P.F.C. Vasconcelos)

**Keywords:** Oropouche virus, molecular epidemiology, genotypes, viral dispersal, viruses, arboviruses, Brazil, research

## Abstract

Oropouche virus (OROV) is the causative agent of Oropouche fever, an urban febrile arboviral disease widespread in South America, with >30 epidemics reported in Brazil and other Latin American countries during 1960–2009. To describe the molecular epidemiology of OROV, we analyzed the entire N gene sequences (small RNA) of 66 strains and 35 partial Gn (medium RNA) and large RNA gene sequences. Distinct patterns of OROV strain clustered according to N, Gn, and large gene sequences, which suggests that each RNA segment had a different evolutionary history and that the classification in genotypes must consider the genetic information for all genetic segments. Finally, time-scale analysis based on the N gene showed that OROV emerged in Brazil ≈223 years ago and that genotype I (based on N gene data) was responsible for the emergence of all other genotypes and for virus dispersal.

*Oropouche virus* (OROV) is one of the most common orthobunyaviruses (family *Bunyaviridae,* genus *Orthobunyavirus*) (*1*) and is the causative agent of Oropouche fever in humans, which is clinically characterized as an acute febrile disease (*2*). The first isolation of OROV was reported in Trinidad and Tobago in 1955, when the virus was isolated from the blood of a febrile patient and from a pool of *Coquillettidia venezuelensis* mosquitoes (*3*). OROV was described in Brazil in 1960, when it was isolated from a sloth (*Bradypus tridactylus*) captured near a forested area during construction of the Belem–Brasilia highway and from a pool of *Ochlerotatus* (*Ochlerotatus*) *serratus* mosquitoes, captured near the same site (*4*).

Since the first isolation of OROV, >30 outbreaks have been reported in Brazil, Peru, Panama, and Trinidad and Tobago during 1960–2009. At least half a million persons are estimated to have been infected (*5**,**6*).

Similar to the genomes of other orthobunyaviruses, the OROV genome comprises 3 single-stranded negative-sense RNA segments—large, medium, and small. The large RNA segment encodes a large protein that has RNA polymerase activity for transcription and replication of genomic RNA segments. The medium segment encodes a precursor polyprotein, which gives rise to the viral surface glycoproteins (Gc and Gn) and to a nonstructural protein NS_M_. The small RNA encodes a structural nucleocapsid (N) protein, as well as a smaller nonstructural protein (NS_S_) in overlapping reading frames (*1*). Studies of the molecular biology of the OROV small RNA segment have suggested its monophyletic origin and the existence of at least 3 genotypes (I, II, and III) (*7*). Recently, genotype III was isolated from a wild vertebrate host (*Callithrix* sp.) in southeastern Brazil, suggesting possible dispersion of the virus to susceptible and populated areas in Brazil (*8*). Further molecular analyses that used OROV strains recovered during outbreaks in Pará State during 2003–2007 demonstrated the association of at least 2 different genotypes (I and II) with Oropouche fever cases in the area (*9**,**10*).

In this study, we describe new information regarding the molecular epidemiology of OROV. This information will help clarify the evolution, dispersal, and genotyping classification of this human pathogen in the Brazilian Amazon region.

## Material and Methods

### Virus Strains

The OROV strains used in this study (Table A1) were relatively low-passage isolates obtained from the virus collection of the Department of Arbovirology and Hemorrhagic Fevers, Evandro Chagas Institute (Ananindeua, Brazil). These strains corresponded to viruses recovered from different hosts and geographic locations that were isolated during 1960–2009.

### Virus Culture and RNA Extraction

Viruses were propagated in monolayer cultures of Vero cells. After 75% of cells showed cytopathic effects, the supernatants of infected cell cultures were collected. RNA extraction was conducted by using a commercial kit (QIAmp Viral RNA Mini Kit; QIAGEN, Valencia, CA, USA) according to the manufacturer’s instructions.

### Reverse Transcription–PCR and Nucleotide Sequencing

For the synthesis and amplification of the OROV small RNA, medium RNA, and large RNA cDNA (cDNA), a 1-step reverse transcription–PCR (RT-PCR) was conducted by using a combination of specific-segment sets of the following primers: small RNA (NORO5: AAAGAGGATCCAATAATGTCAGAGTTCATTT; ORO N3: GTGAATTCCACTATATGCCAATTCCGAATT), medium RNA (Gn15S: GGCAACAAACAGTGACAAT and Gn659R: CTATGTTAACGCACATTGCT), and large RNA (LOROF: CCGAAACAAACAAAAACAAT; and large RNA (LOROF: CCGAAACAAACAAAAACAAT and LOROR: GGATGAGTAAGCAATTCTGG) (*7*). Amplicon lengths were expected to be 693 bp, 644 bp, and 634 bp for small RNA, medium RNA, and large RNA, respectively. The RT-PCR products were visualized onto 1.2% agarose gel stained with ethidium bromide (0.5 μg/mL). Amplicons were sequenced by using the same primers applied for the RT-PCR cycling and the ABI PRISM Dye Terminator Kit (Applied Biosystems, Foster City, CA, USA) by using the dideoxyribonucleotide chain terminator method (*11*). The ABI 3130 capillary automated sequencer (Applied Biosystems) was used to obtain the sequence. Both cDNA strands were sequenced from at least 3 RT-PCR products.

### Sequence Analysis and Phylogeny

Sequences obtained for the N (complete), Gn, and large (partial) genes were first inspected in quality by the SeqMan LaserGene package (DNA STAR, Madison, WI, USA) and then used for multiple sequencing alignments with other OROV sequences available in GenBank (www.ncbi.nlm.nih.gov/genbank). The genetic divergence for each gene was determined by using MEGA4 software (*12*) based on the dataset generated by the alignments. Confidence interval for inclusion into a given phylogenetic group was estimated according to the mean of genetic divergence calculated for the known OROV genotypes (I, II, and III) and used as a criterion for searching other genotype groups.

The phylogenetic analysis was performed by comparing the 66 entire N genes and 36 partial Gn genes and large sequences of Brazilian OROV strains, respectively, with homologous sequences obtained from other OROVs isolated from different regions of Central and South America, periods of time, and source of isolation (Table 1). Phylogenetic trees were constructed by using the neighbor-joining (*13*), maximum-likelihood, and maximum-parsimony methods in the PAUP 4.0 software (*14*) as described (*8*). Bayesian and time-scaled (chronologic) analyses also were conducted as described by Rodrigues et al. (*15*). Sequences obtained from the OROV isolates were deposited in GenBank (Table, GenBank accession numbers of previously sequenced OROV and other Simbu group virus strains; Table A1).

**Table 1:** Percentage of genetic divergence between Oropouche virus phylogenetic groups on the basis of the complete N (small RNA) and partial Gn (medium RNA) and L (large RNA) gene sequences, Brazil*

Gene and group	Divergence among genotypes, %				Mean of genetic divergence intergroup
I	II	III	IV
N gene					
I					
II	3.0 (2.0)				
III	4.4 (3.1)	3.0 (2.0)			
IV	5.3 (3.6)	5.3 (3.6)	6.8 (3.9)		
IV in relation to I, II, and III					5.8 (4.0)
I, II, and III					3.5 (2.3)
I, II, III, and IV					4.6 (3.4)
Gn gene					
I					
II	4.5				
III	7.2	5.6 (3.8)			
III in relation to I and II					5.7 (3.8)
L gene					
I					
II	0.8 (0)				
II in relation to I					0.5 (0)

*Percentages within parenthesis are the amino acid sequence divergences among Oropouche virus strains.

### Evaluation of RNA Segment Topologies

To evaluate the topologies presented by the different RNA segments, we used 36 OROV strains for which all 3 segments were sequenced. The evaluation was performed by using the Kishino-Hasegawa method (*16*), comparing a topology generated for a given RNA segment with the other segments. We considered p values <0.01 significant.

## Results

### Genetic Variability of OROV

The nucleotide sequences obtained for the studied strains were 693 nt (231 aa), 644 nt (214 aa), and 634 nt (211 aa) in length for N, Gn, and large genes, respectively. The multiple sequencing analysis of the new 66 full-length OROV N (small RNA) and for the 36 partial Gn (medium RNA) and large RNA gene sequences showed high nucleotide and amino acid identities (>90%). The mean of genetic divergence among the N gene nucleotide sequence was ≈6.8%. Genetic distances (nucleotide sequence) within the 3 well-established genotypes (I, II, and III) ranged from 3% between genotypes I and II to 4.4% between genotypes I and III (mean 3.5%) and were used as a confidence value for inclusion within a given genotype. On the basis of this criterion, a fourth group was established, and a genetic divergence ranging from 5.3% with genotype I to 6.8% with genotype III (mean 5.8%) was determined. The mean of genetic divergence among the 4 OROV lineages was 4.6% (Table 1).

Regarding the Gn gene nucleotide sequences, the analysis showed values of genetic divergence of 0.9%–9.5% (mean 6.5%). In contrast to the N gene sequences, for the Gn gene partial sequences, 3 lineages were identified, showing an intergroup divergence of 4.5% (between groups I and II) to 7.2% (between groups I and III) (mean 5.7%), which was used as a confidence value for group inclusion or exclusion (Table 1).

For the polymerase gene nucleotide sequences (large RNA), genetic divergence was 0.1%–0.8% (mean 0.5%). Only 2 large RNA segments were distinguished into groups (Table 1).

### Phylogeny and Time-scaled Analysis

**Figure 1:**
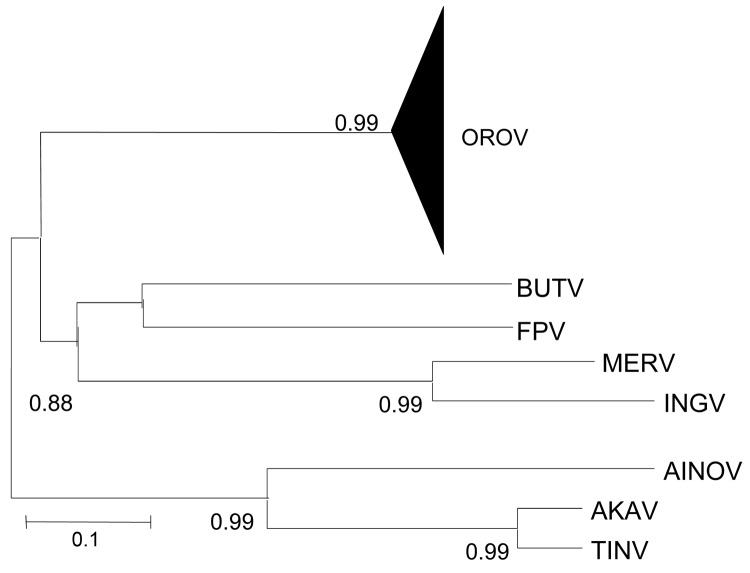
Phylogenetic analysis between Oropouche virus (OROV) (N gene: 693 nt) and homologue sequences of different viruses that belong to the Simbu group. AINOV, Aino virus; AKAV, Akabane virus; TINV, Tinaroo virus; BUTV, Buttonwillow virus; FPV, Facey’s Paddock virus; MERV, Mermet virus; INGV, Ingwavuma virus. The numbers above each main node correspond to bootstrap values for phylogenetic groups. Scale bar indicates 10% genetic divergence.

**Figure 2:**
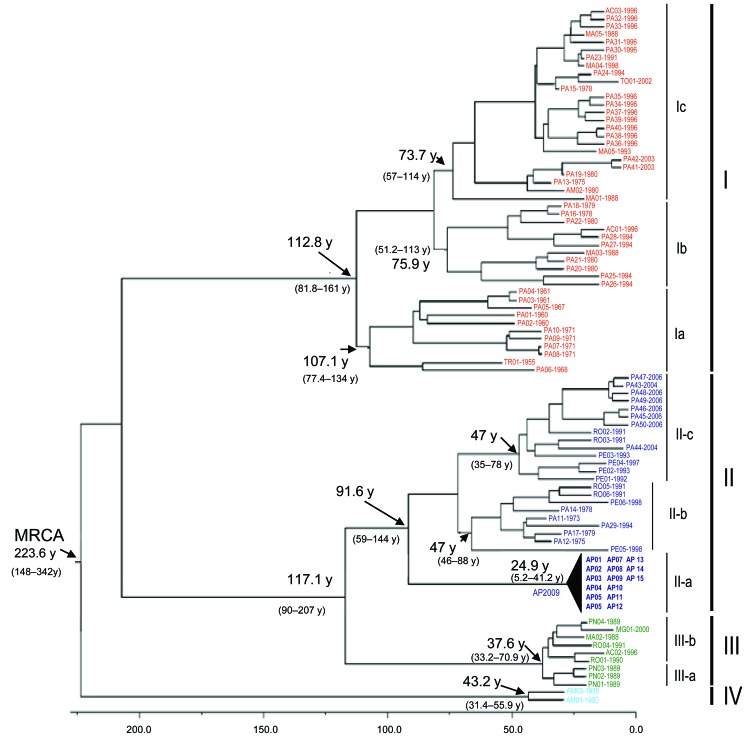
Phylogenetic tree based on the complete nucleotide (nt) sequence of the N gene (693 nt) of 96 Oropouche virus (OROV) strains isolated from different hosts, locations, and periods. The main phylogenetic groups are represented by genotypes I (red), II (dark blue), III (green), and IV (light blue). The values above the main nodes represent the dates of emergence of common ancestors, expressed in years before 2009. The arrows indicate the probable date of emergence of genotypes I, II, III, and IV. Numbers in parentheses are value for 95% highest probability density. Scale bar indicates time scale of molecular dating. MRCA, most recent common ancestor.

Regardless of the method used, the trees were similar in topology, showing high support values (bootstrap, likelihood, or posterior probability values). The Bayesian method showed high support values (>0.90) and was therefore used to represent the final tree. As previously reported (*7**–**10*), the comparative phylogeny that used the entire N gene sequences (96 strains; Table A1) confirmed the monophyletic origin of OROV in comparison with other Simbu group viruses (Figure 1),

The 4 major phylogenetic groups depicted (I–IV) corresponded to 4 distinct genotypes (Figure 2 [Bayesian method]). Genotype I included the Brazilian strains isolated in the states of Acre, Amazonas, Maranhão, Tocantins, and Pará, as well as strains from Trinidad and Tobago. Three subgenotypes were described: Ia, Ib, and Ic (Figure 1). Genotype II grouped strains obtained during outbreaks in the states of Amapá, Pará, and Rondônia in Brazil and the strains from Peru. Three subgenotypes also were assigned to this group (II a, II b, and II c). Genotype III was formed by strains isolated in the Brazilian states of Acre, Minas Gerais, and Rondônia, and the isolates from Panama showing 2 distinct sublineages: the subgenotypes II a and III b. Finally, genotype IV included the Brazilian strains isolated in Amazonas State, Brazil (Figure 2).

Chronologic analysis was used to investigate the emergence period of OROV in the Americas. The nucleotide substitution rate that determined the 96 OROV N gene sequences was 3.7 × 10^–4^ substitutions per site per year and was used to estimate the divergence dates among the strains. The emergence of the most recent common ancestor (MRCA) for OROV in the Americas was estimated to have occurred ≈223 years ago (95% highest probability density [HPD] 148–342 years) from the location where the other parental viruses for the different genotypes (I, II, III, and IV) emerged (Figure 2). The estimated emergence dates suggest that genotype I was the first genotype that emerged ≈112 years ago (95% HPD 95–189 years). Genotype II emerged ≈91 years ago (95% HPD 59–144 years) and originated from strains isolated in the states of Pará and Rondônia, and strains recently isolated in Amapá State, in 2009. Genotype III was estimated to have originated 37 years ago (95% HPD 33–70 years) and probably evolved in Rondônia State 33 years ago (95% HPD 29–58 years), and other Amazonian states, such as Acre and Pará, emerging almost simultaneously in Panama 32 years ago (95% HPD 22–45 years) and, more recently, in Minas Gerais State. Genotype IV emerged in Amazonas State ≈43 years ago (95% HPD 31–56 years; Figure 2).

### Evaluation of RNA Segment Topologies

**Figure 3:**
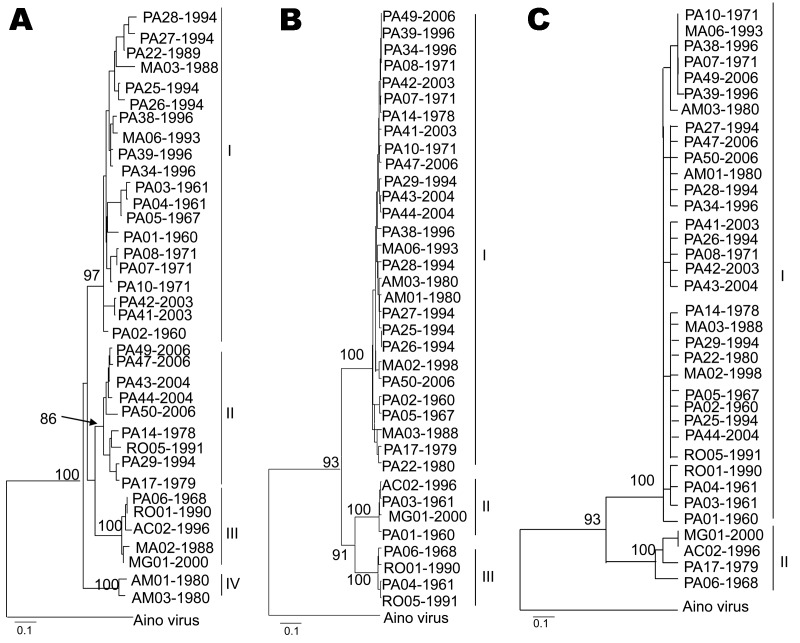
Phylogenetic analysis of 36 Oropouche virus strains: A) N gene (693 nt), B) Gn gene (644 nt), and C) large (L) gene (634 nt), showing different topologies. Bootstrap values obtained by using maximum parsimony and neighbor-joining methods are placed over each main node of the tree corresponding to the phylogenetic groupings. The arrow indicates the exact position of the bootstrap value in the tree. Scale bars indicate 10% nt divergence.

Trees generated from entire N and partial Gn and large gene sequences obtained for 36 OROV strains demonstrated different topologies. By using all phylogenetic methods, we found differences in virus clustering. For the small RNA, 4 distinct groups were identified: group I (20 strains), group II (9 strains), group III (5 strains), and group IV (2 strains). For the medium RNA, 3 groups were assigned and distributed as follows: group I (28 strains), group II (4 strains), and group III (4 strains). The large RNA depicted only 2 major groups, including 32 strains in group I and 4 strains in group II (Figure 3). Maximum likelihood was used to analyze these competing small, medium, and large segment topologies by using the Kishino-Hasegawa test. Sequence evolution models were optimized by applying all genome segments and using the competing topologies. Regardless of which model was selected, each topology generated by using maximum parsimony and neighbor-joining methods with a given genome segment was significantly more likely than the competing topology generated by using the other genome segment (likelihood probability between S and M topologies = 0.00005623; likelihood probability between S and L topologies = 0.000354664; likelihood probability between M and L topologies = 0.00043154; p<0.001).

### Geographic Dispersion of OROV Genotypes

**Figure 4:**
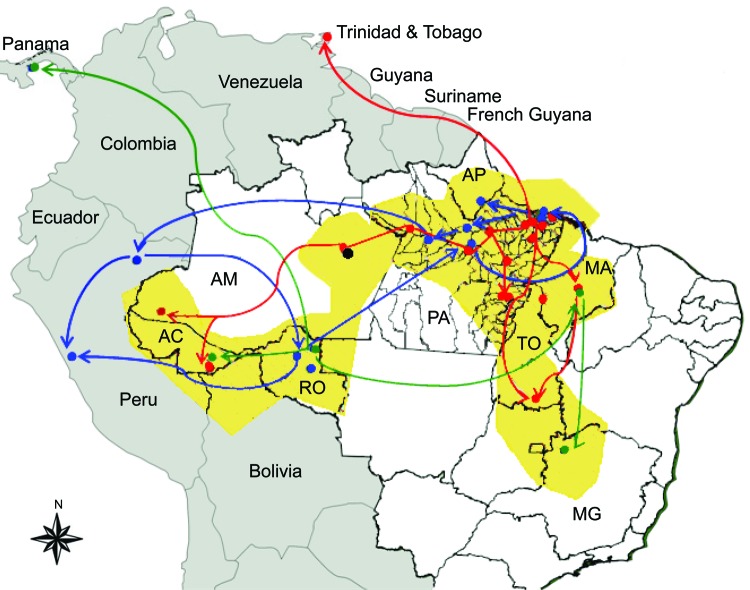
Geographic dispersion of Oropouche virus (OROV) genotypes in South America during 1955–2009 based on data from the N gene. Yellow shading, coverage area of OROV in Brazil; red line, dispersion route for genotype I; blue line, dispersion route for genotype II; green line, dispersion route for genotype III; black dot, genotype IV. AC, Acre; AP, Amapá; AM, Amazonas; MA, Maranhão; MG, Minas Gerais; PA, Pará; RO, Rondônia, TO, Tocantins.

On the basis of results obtained for the N gene data by time-scaled analysis (evolutionary rate and emergence date) and epidemiologic data association (date and place of isolation), the possible dispersal event could be predicted for the distinct OROV genotypes in the Americas (Figure 4). Genotype I (dispersion route in red), originally isolated in Brazil in the municipality of Ipixuna, Pará State (BR 010 Highway, km 94), possibly dispersed continuously toward distinct directions: initially to several municipalities in western Pará, and simultaneously in Trinidad and Tobago. Later, genotype I moved toward the states of Amazonas and Acre and, more recently, to the eastern Amazon region including Pará, Maranhão, and Tocantins States. Genotype II (dispersion route in dark blue), apparently emerged simultaneously in the states of Amapá, Pará, and Rondônia, as well as in Peru, and dispersed in these places, emerging in the municipality of Mazagão, Amapá State, in 2009. Genotype III (dispersion route in green), emerged in Rondônia State, moving toward Panama and the states of Acre and Maranhão. From Maranhão, a new route led genotype III to the Minas Gerais State. Genotype IV (black dot in Manaus), apparently more ancient than genotype III, emerged in the city of Manaus, Amazonas State, and it has not apparently dispersed from there (Figure 4).

## Discussion

The molecular epidemiology of OROV has been extensively studied on the basis of genetic data generated for the small RNA segment, and the data have provided information about the genetic diversity of OROV and geographic distribution in countries in which the virus is endemic, such as Brazil, Peru, and Trinidad and Tobago (*7**–**10**,**17*). The analysis of additional 66 gene sequences of the entire N and partial Gn and Gc provided a better understanding of the molecular epidemiology of OROV in Brazil. In our analysis, distinct phylogenetic groups were observed when the different RNA segments were analyzed. In case of the small RNA, 4 major groups were found, including the 3 genotypes previously described (*7**–**10**,**17*). Although a fourth genetic lineage has been well established by the small RNA phylogeny (strains AM 01 and AM 03), the topologies depicted by the medium RNA and large RNA sequences did not support this result. Maximum likelihood analyses were used to test these competing small, medium, and large segment topologies by using the Kishino-Hasegawa test. Evolution models were optimized for all 3 genome segment sequences and by using the competing topologies. Regardless of which model was selected, each topology generated by using maximum parsimony and neighbor-joining methods with a given RNA segment was significantly more likely than the competing topology generated by the other genome segment (p<0.001) (Figure 3). These results ensured that the testing topologies obtained for each RNA segment differed significantly, which suggests that each OROV RNA segment had a different evolutionary history and probably contributes to the genetic variability of the virus.

The assessment of additional genetic data for the small RNA segment contributed substantially to the understanding of the emergence of the virus, geographic distribution, and dispersal events. On the basis of chronologic dating of the N gene, epidemiologic data, and lineage definition (genotypes I–IV), we were able to elucidate the possible origin of OROV in the Americas (Figure 2, Figure 4). In contrast to information about the event in Trinidad and Tobago in 1955 that was associated with the first description of the Oropouche fever case, molecular data provided by the small RNA sequences indicated that OROV emerged in South America, more precisely in Pará State (strains PA 01–PA 05) in northern Brazil, ≈89 years ago, and then in Trinidad and Tobago probably through humans carrying the virus during the viremic phase or through illegal shipment of wild animals, as has been suggested for yellow fever virus (*18*).

The dispersal history of OROV strains is initially associated with genotype I, more precisely with the subgenotype Ia, isolated from wild animals and humans during epidemics in Pará State, during the 1960s–1970s. Their dispersion routes were simultaneously west to east in the Amazon toward Acre State (subgenotype Ib) from 1988 to 1994 and, more recently, in a vast area in Pará State and in Manaus, Amazonas State, at the end of the 1990s and the beginning of the 2000s.

Regarding genotype II, the most probable origins were in eastern Pará (Porto de Moz) toward Iquitos, Peru (subgenotype IIb), and from Iquitos toward Ariquemes, Rondônia State (subgenotype IIc), where the virus probably then dispersed to Madre de Dios in Peru and to Pará State. The origin of subgenotype IIa, which is represented by the strains recently associated with the epidemic in Mazagão, Amapá State, in the beginning of 2009 (P.F.C. Vasconcelos et al., pers. comm.) is probably related to a common ancestor that evolved independently from other subgenotypes (IIb and IIc) over time and probably emerged in the Amazon ≈24 years ago.

The existence of genetic data for a single genotype III Brazilian strain isolated in Minas Gerais State, southeastern Brazil (*8*), limited our ability to study its origin and evolutionary aspects. With the identification of other genotype III strains in Brazil, isolated in the states of Rondônia (Ariquemes and Machadinho d’Oeste), Acre (Xapuri), and Maranhão (Porto Franco), we were able to make inferences about the most possible dispersion route. In fact, it constitutes a complex dynamics of evolutionary origin between subgenotypes IIIa (predominantly from Brazil) and IIIb (predominantly from Panama). In this context, genotype III probably originated from the sublineage IIIa, which was isolated in Ariquemes, Rondônia State, from which the sublineage IIIb ancestor has segregated independently, leading to the emergence of strains in Chame and San Miguelito, Panama.

In a more detailed view, the subgenotype IIIa found in Ariquemes, Rondônia State, had its initial dispersion to a neighboring municipality (Machadinho d’Oeste), subsequently to Porto Franco in Maranhão State, and finally to Arinos, Minas Gerais State. Although Minas Gerais State is geographically distant from the official OROV-endemic area, the virus may have been introduced through Maranhão State by the intense traffic of humans from Maranhão to other states and regions in Brazil.

In Minas Gerais, OROV has been maintained in silent cycles, probably because of inadequate epidemiologic conditions, such as the high density of *Culicoides paraensis* mosquitoes in urban areas, a limiting factor for an epidemic cycle deflagration. Furthermore, the sporadic detection of OROV was recently reported in Acre State (*19*); these reports confirmed that the virus actually circulates silently in the Brazilian Amazon, as suggested by Azevedo et al. (*9*), and can be transported by viremic patients and human carriers of subclinical illness from region to region within the country. This approach should result in stronger data when new isolates are sequenced in other OROV-endemic countries because limited information about dispersal of OROV in Peru, Panama, and Trinidad and Tobago does not infer a more robust analysis.

In conclusion, even with the limited data obtained in this study from other OROV-endemic countries, we were able to reach a more complete understanding of the molecular epidemiology of the virus, and we provided evidence of which distinct genes (N, Gn/Gc, and L) are under different selective evolutionary pressures in nature. We also observed the great genetic diversity of OROV, the description of a new genotype IV, the complex dynamics of evolution, and viral dispersal. Finally, our findings suggest the necessity of obtaining genetic data regarding full-length sequencing of different OROV strains (medium and large segments) to elucidate the correct genotype classification and to improve the molecular diagnostics of this human pathogen in Latin America.

## Appendix

**Table A1:** Oropouche virus strains used for phylogenetic analysis and time-scaled analysis*

Label	Strains	Source of isolation	Year	Place of isolation†	GenBank accession no.
TR01	TRVL 9760	Human	1955	Sangre Grande, Trinidad and Tobago	S: AF164531
PA01	AR 19886	*Ochlerotatus serratus* mosquitoes	1960	BR 14 KM 94 (Ipixuna), Pará	S: HM470107; M: HQ830373; L: HQ830408
PA02	AN 19991	*Bradypus tridactylus*	1960	São Miguel do Guamá, Pará	S: AF164532; M: AF 441119; L: AF 484424
PA03	H 29086	Human	1961	Belém, Pará	S: HM470108; M: HQ830374; L: HQ830409
PA04	H 29090	Human	1961	Belém, Pará	S: HM470109; M: HQ830375; L: HQ830410
PA 05	H 121293	Human	1967	Bragança, Pará	S: HM470110; M: HQ830376; L: HQ830411
PA06	AR 136921	*Culex quinquefasciatus* mosquitoes	1968	Belém, Pará	S: HQ830443; M: HQ830377; L: HQ830412
PA07	AN 206119	*B. trydactilus*	1971	Maracanã, Pará	S: AY993909; M: HQ830378; L: HQ830413
PA08	AN 208402	*B. trydactilus*	1971	Maracanã, Pará	S:AY993910; M: HQ830379; L: HQ830414
PA09	AN 208819	*B. trydactilus*	1971	Maracanã, Pará	S:AY993911; M: HQ830380; L: HQ830415
PA10	AN 208823	*B. trydactilus*	1971	Maracanã, Pará	S: AY993912
PA11	H 244576	Human	1973	Belém, Pará	S: HQ830444
PA12	H 271708	Human	1975	Santarém, Pará	S: HQ830445
PA13	AR 271815		1975	Santarém, Pará	S: AF164533
PA14	H 355173	Human	1978	Ananindeua, Pará	S: HM470114; M: HQ830381; L: HQ830416
PA15	H 355186	Human	1978	Tomé-Açu, Pará	S: HQ830446
PA16	H 356898	Human	1978	Belém, Pará	S: HQ830447
PA17	AR 366927	*Culicoides paraensis* mosquitoes	1979	Belém, Pará	S: HQ830448; M: HQ830382; L: HQ830417
PA18	H 366781	Human	1979	Belém, Pará	S: HQ830449
PA19	H 381114	Human	1980	Belém, Pará	S: AF164435
PA20	H 384192	Human	1980	Portel, Pará	S: HQ830450
PA21	H 384193	Human	1980	Portel, Pará	S: HQ830451
PA22	H 385591	Human	1980	Belém, Pará	S: HQ830452; M: HQ830383; L: HQ830418
AM01	H 389865	Human	1980	Manaus, Amazonas	S: HQ830453; M: HQ830384; L: HQ830419
AM02	H 390233	Human	1980	Manaus, Amazonas	S: AF154536
AM03	H 390242	Human	1980	Manaus, Amazonas	S: HQ830454; M: HQ830385; L: HQ830420
MA01	AR 473358		1988	Porto Franco, Maranhão	S: AF164539
MA02	H 472433	Human	1988	Porto Franco, Maranhão	S: HQ830455; M: HQ830386; L: HQ830421
MA03	H 472435	Human	1988	Porto Franco, Maranhão	S: HQ830456; M: HQ830387; L: HQ830422
MA04	H 472200	Human	1988	Porto Franco, Maranhão	S: AF154537
MA05	H 472204	Human	1988	Porto Franco, Maranhão	S: AF164538
PA23	H 475248	Human	1988	Tucurui, Pará	S: AF164540
PN01	GML 444477	Human	1989	Chame, Panama	S: AF164555
PN02	GML 444911	Human	1989	Chame, Panama	S: AF164556
PN03	GML 445252	Human	1989	San Miguelito, Panama	S: AF164557
PN04	GML 450093	Human	1989	Chilibre, Panama	S: AF164558
RO01	H 498913	Human	1990	Machadinho D'Oeste, Rondônia	S: HQ830457; M: HQ830388; L: HQ830423
RO02	H 505442	Human	1991	Ouro Preto D'Oeste, Rondônia	S: AF164542
RO03	H 505663	Human	1991	Ariquemes, Rondônia	S: AF164543
RO04	H 505764	Human	1991	Ariquemes, Rondônia	S: HQ830458
RO05	H 505768	Human	1991	Ariquemes, Rondônia	S: HQ830459; M: HQ830389; L: HQ830424
RO06	H 505805	Human	1991	Ariquemes, Rondônia	S: HQ830460
PA24	H 504514	Human	1991	Santa Izabel, Pará	AF164541
PE01	IQT 1690		1992	Iquitos, Peru	AF164549
MA06	H 521086	Human	1993	Barra do Corda, Maranhão	S:AY704559; M: HQ830390; L: HQ830425
PE02	MD O 23	Human	1993	Madre de Dios, Peru	S: AF164550
PE03	DE I209	Human	1993	Iquito, Peru	S: AF164551
PA25	H 532314	Human	1994	Serra Pelada, Pará	S: HQ830461; M: HQ830391; L: HQ830426
PA26	H 532422	Human	1994	Serra Pelada, Pará	S: HQ830462; M: HQ830392; L: HQ830427
PA27	H 532490	Human	1994	Serra Pelada, Pará	S: HQ830463; M: HQ830393; L: HQ830428
PA28	H 532500	Human	1994	Serra Pelada, Pará	S: HQ830464; M: HQ830394; L: HQ830429
PA29	H 541140	Human	1994	Altamira, Pará	S: HM470126; M: HQ830395; L: HQ830430
PA30	H 541863	Human	1996	Vitória do Xingu, Pará	S: AF164544
PA31	H 544552	Human	1996	Altamira, Pará	S: AF164546
PA32	H 543033	Human	1996	Oriximiná, Pará	S: AF164545
AC01	H 543091	Human	1996	Xapuri, Acre	S: HQ830465
AC02	H 543100	Human	1996	Xapuri, Acre	S: HQ830466; M: HQ830396; L: HQ830431
AC03	H 543087	Human	1996	Xapuri, Acre	S: AF164547
PA33	H 543618	Human	1996	Oriximina, Pará	S: AF164548
PA34	H 543629	Human	1996	Oriximina, Pará	S: HQ830467; M: HQ830397; L: HQ830432
PA35	H 543638	Human	1996	Oriximina, Pará	S: HQ830468
PA36	H 543639	Human	1996	Oriximina, Pará	S: HQ830469
PA37	H 543733	Human	1996	Oriximina, Pará	S: AY704560
PA38	H 543760	Human	1996	Oriximina, Pará	S: HQ830470; M: HQ830398; L: HQ830433
PA39	H 543857	Human	1996	Oriximina, Pará	S: HQ830471; M: HQ830399; L: HQ830434
PA40	H 543880	Human	1996	Oriximina, Pará	S: HQ830472
PE04	IQT 4083		1997	Iquitos, Peru	S: AF164552
PE05	IQT 1–812		1998	Iquitos, Peru	S: AF164553
PE06	IQT 7085		1998	Iquitos, Peru	S: AF164554
MG01	AN 622998	*Callitrhix* sp.monkeys	2000	Arinos, Minas Gerais	S: AY117135; M: HQ830401; L: HQ830436
TO01	H 622544	Human	2002	Paranã, Tocantins	S: EF467368
PA41	H 669314	Human	2003	Parauapebas, Pará	S: EF467370; M: HQ830400; L: HQ830435
PA42	H 669315	Human	2003	Parauapebas, Pará	S: EF467369; M: HQ830402; L: HQ830437
PA43	H 682426	Human	2004	Porto de Moz, Pará	S: EF467371; M: HQ830403; L: HQ830438
PA44	H 682431	Human	2004	Porto de Moz, Pará	S: EF467372; M: HQ830404; L: HQ830439
PA45	H 706890	Human	2006	Igarapé Açu, Pará	S: HQ830473
PA46	H 706893	Human	2006	Igarapé Açu, Pará	S: HQ830474
PA47	H 708139	Human	2006	Magalhães Barata, Pará	S: HQ830475; M: HQ830405; L: HQ830440
PA48	H 707157	Human	2006	Maracanã, Pará	S: HQ830476
PA49	H 707287	Human	2006	Magalhães Barata, Pará	S: HM470137; M: HQ830406; L: HQ830441
PA50	H 708717	Human	2006	Maracanã, Pará	S: HQ830477; M: HQ830407; L: HQ830442
AP01	H 758687	Human	2009	Mazagão, Amapá	S: HQ830478
AP02	H 758669	Human	2009	Mazagão, Amapá	S: HQ830479
AP03	H 759525	Human	2009	Mazagão, Amapá	S: HQ830480
AP04	H759541	Human	2009	Mazagão, Amapá	S: HQ830481
AP05	H 759531	Human	2009	Mazagão, Amapá	S: HQ830482
AP06	H 759558	Human	2009	Mazagão, Amapá	S: HQ830483
AP07	H 759038	Human	2009	Mazagão, Amapá	S: HQ830484
AP08	H 759562	Human	2009	Mazagão, Amapá	S: HQ830485
AP09	H 759018	Human	2009	Mazagão, Amapá	S: HQ830486
AP10	H 759023	Human	2009	Mazagão, Amapá	S: HQ830487
AP11	H 759041	Human	2009	Mazagão, Amapá	S: HQ830488
AP12	H 759042	Human	2009	Mazagão, Amapá	S: HQ830489
AP13	H 759043	Human	2009	Mazagão, Amapá	S: HQ830490
AP14	H 759044	Human	2009	Mazagão, Amapá	S: HQ830491
AP15	H 759483	Human	2009	Mazagão, Amapá	S: HQ830492

*TRVL, Trinidad Virus Laboratory; S, small RNA; AR: arthropod; M, medium RNA; L, large RNA; H: human; An: animal; GML, Gorgas Memorial Laboratory; IQT, Iquitos; MD, Madre de Dios; DE, OROV strain from Iquitos. †All locations in Brazil except as indicated.
